# The effect of ginger extract on cisplatin-induced acute anorexia in rats

**DOI:** 10.3389/fphar.2023.1267254

**Published:** 2023-11-09

**Authors:** Hyeonah Kim, Keun-Tae Park, Heejoon Jo, Yuchan Shin, Geehoon Chung, Seong-Gyu Ko, Young-Ho Jin, Woojin Kim

**Affiliations:** ^1^ Department of Physiology, College of Korean Medicine, Kyung Hee University, Seoul, Republic of Korea; ^2^ Korean Medicine-Based Drug Repositioning Cancer Research Center, College of Korean Medicine, Kyung Hee University, Seoul, Republic of Korea; ^3^ Department of Physiology, College of Medicine, Kyung Hee University, Seoul, Republic of Korea

**Keywords:** anorexia, chemotherapy-induced anorexia, cisplatin, ginger, nodose ganglion, serotonin

## Abstract

Cisplatin is a platinum-based chemotherapeutic agent widely used to treat various cancers. However, several side effects have been reported in treated patients. Among these, acute anorexia is one of the most severe secondary effects. In this study, a single oral administration of 100 or 500 mg/kg ginger extract (GE) significantly alleviated the cisplatin-induced decrease in food intake in rats. However, these body weight and water intake decreases were reversed in the 100 mg/kg group rats. To elucidate the underlying mechanism of action, serotonin (5-HT) and 5-HT_2C_, _3A,_ and _4_ receptors in the nodose ganglion of the vagus nerve were investigated. The results showed that cisplatin-induced increases in serotonin levels in both the blood and nodose ganglion tissues were significantly decreased by100 and 500 mg/kg of GE administration. On 5-HT receptors, 5-HT_3A_ and _4_, but not _2C_ receptors, were affected by cisplatin, and GE 100 and 500 mg/kg succeeded in downregulating the evoked upregulated gene of these receptors. Protein expression of 5-HT_3A_ and _4_ receptors were also reduced in the 100 mg/kg group. Furthermore, the injection of 5-HT_3A,_ and _4_ receptors antagonists (palonostron, 0.1 mg/kg, i.p.; piboserod, 1 mg/kg, i.p., respectively) in cisplatin treated rats prevented the decrease in food intake. Using high-performance liquid chromatography (HPLC) analysis, [6]-gingerol and [6]-shogaol were identified and quantified as the major components of GE, comprising 4.12% and 2.15% of the GE, respectively. Although [6]-gingerol or [6]-shogaol alone failed to alleviate the evoked anorexia, when treated together, the effect was significant on the cisplatin-induced decrease in food intake. These results show that GE can be considered a treatment option to alleviate cisplatin-induced anorexia.

## 1 Introduction

Cisplatin (cis-diamminedichloroplatinum II) is currently one of the most effective antitumor drugs (Brown et al., 2019). First discovered by Michele Peyrone in 1844, cisplatin was approved by the Food and Drug Administration (FDA) in 1979 to treat solid tumors (Muggia, 2009). Since its approval, it has been used to treat various cancers, including ovarian, testicular, cervical, and bladder cancers (Prestayko et al., 1979). Cisplatin causes deoxyribose nucleic acid (DNA) damage in cancer cells, regulates ataxia telangiectasia mutation (ATM), and activates apoptosis-induced p53 through several signaling pathways to treat cancers (De Laurenzi and Melino, 2000; Shimodaira et al., 2003). However, cisplatin can also induce side effects such as nephrotoxicity, ototoxicity, and gastrointestinal toxicity (Prestayko et al., 1979). Anorexia is an eating disorder characterized by the loss of appetite, including the early onset of satiety (Shiomi et al., 2018). The treatment of anorexia in chemotherapy-treated patients is important because it can ultimately lead to cachexia (Plata-Salamán, 1996). Furthermore, anorexia not only adversely affects patients’ quality of life (QOL), but can also lead to failure of chemotherapy treatment, as proper nutrition intake could become difficult (Mercadante, 1996). Therefore, controlling cisplatin-induced eating disorders is important for improving the QOL and treating cancer patients (Ballatori and Roila, 2003). Drugs such as ondansetron and dexamethasone are used to attenuate anorexia (Aapro and Alberts, 1981); however, side effects such as headaches, diarrhea and pain have also been reported in the treated patients (Tramer et al., 1997; Bordag et al., 2015). Therefore, efforts are needed to identify novel therapeutic agents.

The involvement of serotonin (5-Hydroxytryptamine; 5-HT) has been reported as a pathological mechanism of anorexia. Following cisplatin administration, 5-HT increases in multiple parts of the brain, such as the hippocampus, hypothalamus, and medulla oblongata (Liu et al., 2003). Moreover, 5-HT receptors, especially 5-HT_2_, _3_, and _4_ receptors, have been reported to play a critical role in anorexia. When 5-HT_2C_ receptor agonists are administered intraperitoneally (i.p.) to rats, food intake is significantly reduced (Hattori et al., 2013). Additionally, 5-HT_2C_ receptor lacking rats showed hyperphagia and obesity (Nonogaki et al., 1998). In addition, intraperitoneal injection of the 5-HT_3_ receptor antagonist granisetron or ondansetron inhibited the delayed gastric discharge induced by cisplatin in rats (Hattori et al., 2013). The role of 5-HT_4_ receptor has also been reported (Tonini, 1995; Yamakuni et al., 2000; Horikoshi et al., 2001), as vomiting occurs when 5-methoxy tryptamine (5-MT), a 5-HT_4_ receptor agonist, is administered orally to dogs (Fukui et al., 1994). These results demonstrate that 5-HT receptors may play a critical role in anorexia.

We have previously reported the effect of ginger extract (GE) on chemotherapy-induced neuropathic pain. Orally administered ginger significantly alleviates pain by modulating spinal 5-HT receptors (Lee et al., 2021). Ginger, the rhizome of *Zingiber officinale* Roscoe, is a spice widely used worldwide (Kubra and Rao, 2012), and its therapeutic effects have been reported in several studies (Al-Awwadi, 2017). Moreover, it was also shown to be effective against various gastrointestinal diseases (Srinivasan, 2017; Nikkhah Bodagh et al., 2019). In an animal model of acetic acid-induced irritable bowel syndrome, oral administration of 50–100 mg/kg GE successfully alleviated symptoms (Zhang et al., 2020). Furthermore, in a clinical trial, patients with functional dyspepsia received ginger and artichoke leaf extracts for 4 weeks every day, and functional dyspepsia symptoms, such as nausea, epigastric bloating, and pain, improved compared to placebo (Giacosa et al., 2015). Although some studies have reported the effect of [6]-gingerol, one of the main components of ginger, on chemotherapy-induced nausea and vomiting (Qian et al., 2009; Cheng et al., 2020; Tian et al., 2020), the effect of whole GE against cisplatin-induced acute anorexia has never been assessed.

In the present study, we demonstrated the effects of GE against cisplatin-induced anorexia. Second, the roles of the 5-HT and its receptors (i.e., 5-HT_2C, 3A,_ and _4_ receptors) in the nodose ganglion were assessed after cisplatin and GE treatment. Finally, quantification and identification of [6]-gingerol and [6]-shogaol in the GE have been assessed alongside with their effects against cisplatin-induced acute anorexia in rats.

## 2 Materials and methods

### 2.1 Animals

Male Sprague-Dawley (SD) rats (body weight 180–200 g, 6 weeks old) purchased from Daehan Bio Link (Chungbuk, Korea) were used in this study. They were maintained under specific pathogen-free animal center and housed in cages under environmentally controlled conditions (23°C ± 2°C, 65% ± 5% humidity, 12 h light and 12 h dark cycle). All experiments were approved by Kyung Hee University Animal Care and Use Committee (KHUASP-22-106) on 22nd March 2022.

### 2.2 Drug administrations

To induced feeding disorder, cisplatin (Sigma-Aldrich, MO, United States) was dissolved in 50 mL of normal saline (NS) to a concentration of 0.5 mg/mL and was injected intraperitoneally in rats to a dose of 2 mg/kg or 6 mg/kg. The administration volume was 2.4 ml [6]-gingerol and [6]-shogaol (FUJIFILM Wako Pure Chemical Corporation, Osaka, Japan) were dissolved in 10% dimethyl sulfoxide (DMSO) and administered intraperitoneally. The administration volume was 1 mL. Palonosetron (Sigma-Aldrich, MO, United States) was dissolved in NS to a concentration of 0.02 mg/mL. Piboserod (MedChem Express, United States) was primarily dissolved in 1 mL of 10% DMSO and added NS to a concentration of 0.2 mg/mL. Both palonosetron and piboserod were administered intraperitoneally with a volume of 1 mL.

### 2.3 Ginger extract (GE) preparation

*Zingiber officinale* Roscoe (ginger) was purchased from Teageukin (Gyungbuk, Korea). 600 g of ginger was extracted with 1 L of 80% ethanol at room temperature for 72 h. These extracts were filtered by using a filter paper (Advantec, Tokyo, Japan). Subsequently, the extracts were evaporated by using a rotary evaporator (Tokyo Rikakikai Co. Ltd, Tokyo, Japan) at 60°C and freeze-dried (Gyrozen Co. Ltd, Inchon, Korea) overnight. As a result, 22.42 g of dried extract was obtained, and the extraction yield was 5.6%. The dried powder was dissolved in distilled water (DW) for oral administration in rats. The voucher specimen number was deposited as KWJ-0001.

### 2.4 Experimental protocols

**FIGURE 1:**
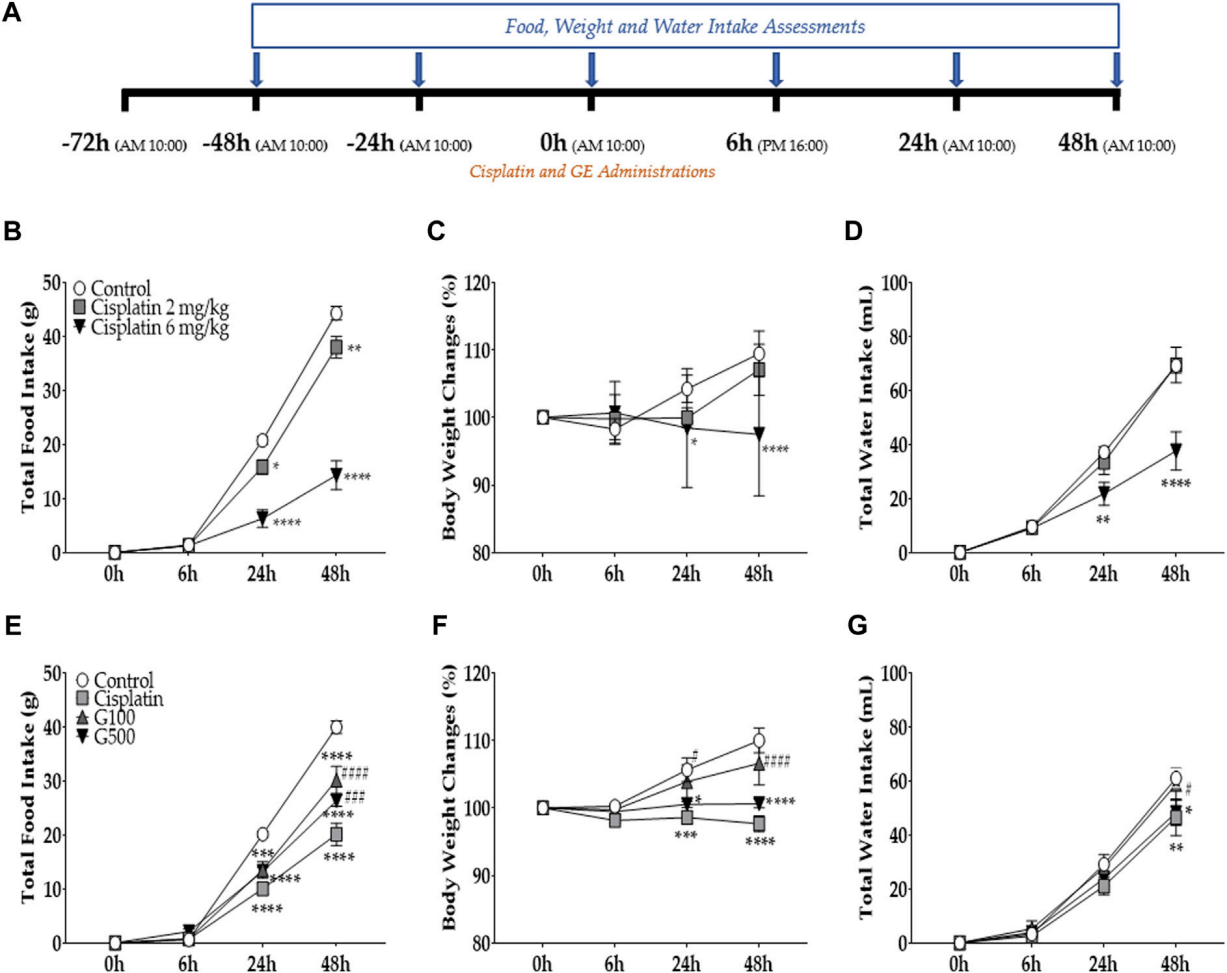
The effect of cisplatin on total food intake, body weight changes, and water intake in rats. And the effect of ginger extract (GE) on cisplatin-induced anorexia in rats. Cisplatin (2 mg/kg and 6 mg/kg) was injected intraperitoneally to induce acute anorectic effect. The amount of food consumed **(B)**, body weight changes **(C)**, and total water intake **(D)** were measured at 6h, 24h, and 48 h time points after single cisplatin injection **(A)**. Cisplatin(6 mg/kg) and GE were administered intraperitoneally and orally respectively. Total intake of foods **(E)**, changes in body weight **(F)**, and water intake **(G)** were assessed. A 6 mg/kg dose of cisplatin was administered intraperitoneal in three groups (i.e., cisplatin, G100, and G500). Control group rats received normal saline (i.p.) and DW (p.o) as a control to cisplatin and GE, respectively. Cisplatin group (Cisplatin) was treated with DW (p.o.) as a control to GE. G100; ginger extract 100 mg/kg, G500; ginger extract 500 mg/kg. Data are presented as the mean ± standard error of the mean (SEM). *N* = 10 each group. * *p* < 0.05, ** *p* < 0.01, *** *p* < 0.001, **** *p* < 0.0001 vs. Control, # *p* < 0.05, ### *p* < 0.001, #### *p* < 0.0001 vs. Cisplatin, with two-way ANOVA followed by Tukey’s post-test for multiple comparisons.

All rats were housed to adapt to metabolic cage (Jeungdo bio and plant co. LTD, Seoul, Korea) prior to the experiments for more than 1 week. For grouping, the food consumption of each rats were measured every 24 h for 72 h and rats were divided into three to five groups based on data obtained for 72 h (Figure 1A). Cisplatin (2 mg/kg and 6 mg/kg), [6]-gingerol (4.12 mg/kg), [6]-shogaol (2.15 mg/kg), palonosetron (0.1 mg/kg), piboserod (1 mg/kg) were injected intraperitoneally and GE (100 mg/kg and 500 mg/kg) was administered orally. NS, DW and 10% DMSO were given as control to cisplatin, GE, and [6]-gingerol, [6]-shogaol in rats, respectively. To assess the effect of cisplatin and GE administration, food and water intake and body weight were measured at 6, 24, and 48 h time point after cisplatin, GE, [6]-gingerol, [6]-shogaol, palonosetron and piboserod treatments. GE, [6]-gingerol, [6]-shogaol, palonosetron and piboserod were injected almost simultaneously with cisplatin. Cisplatin was injected first and subsequently GE, [6]-gingerol, [6]-shogaol, palonosetron or piboserod were given orally or intraperitoneally using different syringes. Once all the experiments were conducted, rats were sacrificed by inhalation of isoflurane. The left and right nodose ganglia and blood serum were removed and stored at −80°C for further analysis.

### 2.5 Enzyme-linked immunosorbent assay (ELISA)

The blood samples were collected via cardiac puncture under isoflurane anesthesia. The serum samples were separated via centrifugation at 13,000 rpm for 20 min at 4°C. After centrifugation, the supernatant was collected. All serum samples were stored at −80°C until analysis. Nodose ganglia was homogenized with radioimmunoprecipitation (RIPA) buffer (Thermo Fisher Scientific, MA, United States) and phosphatase inhibitor cocktail (Thermo Fisher Scientific, MA, United States). The homogeneous tissues were left in ice for 15 min and centrifuged for 20 min at 13,000rpm at 4°C. The supernatants were collected. The 5-HT levels in the serum and nodose ganglia were measured using a Serotonin Research ELISA (LDN, Nordhorn, Germany) according to the manufacturer’s instructions.

### 2.6 Quantitative real-time polymerase chain reaction (qRT-PCR)

Total ribonucleic acid (RNA) was isolated from nodose ganglia and extracted by using easy-BLUE™ Total RNA Extraction Kit (iNtRON Biotechnology, Seongnam, Korea) according to the manufacturer’s protocol. The concentration of RNA was measured by NanoDrop ND-1000 Spectrophotometer (Thermo Scientific, DE, United States), and cDNA was synthesized with Maxime™ RT-PCR PreMix (iNtRON Biotechnology, Seongnam, Korea). We synthesized cDNA synthesis through quantified mRNA through nanodrops. Synthesis Protocol was 1 h at 45°C, 5 min at 95°C, and 1 h at 4°C. The qRT-PCR was performed by using SensiFAST SYBR No-ROX kit (Meridian bioscience, Cincinnati, OH, United States) and CFX Connect Real-Time PCR Detection System (Bio-Rad, CA, United States). Primer sequence followed Table 1. The protocol configured 30 s at 95°C and cycled 45 times. The cycle configuration is 10 s at 95°C followed by 55°C 15 s and 72°C 10 s. The values were calculated through 2^−ΔΔCT^.

**TABLE 1:** PCR primer sequences for PCR analysis.

Type	Sequence
*GAPDH* (Forward)	5′-TGG​TGA​AGG​TCG​GTG​TGA​AC-3′
*GAPDH* (Reverse)	5′-CGA​CAT​ACT​CAG​CAC​CAG​CA-3′
*HTR2C* (Forward)	5′-GAC​TGA​GGG​ACG​AAA​GCA​AAG-3′
*HTR2C* (Reverse)	5′-GAA​GGA​CCC​GAT​GAG​AAC​GA-3′
*HTR3A* (Forward)	5′-GTG​ACC​GCC​TGT​AGC​CTT​GA-3′
*HTR3A* (Reverse)	5′-GAT​GCT​CTT​GTC​CGA​CCT​CA-3′
*HTR4* (Forward)	5′-TGC​CTT​CCT​TAT​CAT​CCT​CTG​C-3′
*HTR4* (Reverse)	5′-CAC​CAC​ATT​CCA​CTG​TAT​CCC​T-3′

### 2.7 Western blot

As mentioned above, nodose ganglia were extracted by centrifugation after homogenization. The supernatants were analyzed using bradford protein analysis (BIO-RAD, CA, United States). Protein samples were loaded in 10% Tris-glycine sodium dodecyl sulfate-polyacrylamide gel followed by electrophoresis and then transferred to the nitrocellulose membrane using a Trans-Blot Turbo Transfer Pack (BIO-RAD, CA, United States). The membrane was blocked for 1 h with 5% skim milk in 0.05% Tris Buffered Saline with Tween 20 (TBS-T). Subsequently, the primary antibody was incubated by adding antibody (β-actin, Invitrogen, 1:2000/5-HT_3A_ and _4_ receptors, Novus Biologicals, 1:1000) to 5% skim milk overnight at 4°C. Secondary antibody was incubated for 1 h with Goat anti-rabbit IgG (H + L) HRP antibody (Invitrogen, 1:2000) in 5% skim milk at room temperature. The band was detected using enhanced chemiluminescence (ECL) solution (Dong-in bio, Seoul, Korea) and imaged with Davinch. Image was quantified by using image J. 5-HT_3A_ and _4_ receptors bands were normalized using the amount of β-actin. Full scan images of the entire original gels are submitted as Supplementary Material S4–S6.

### 2.8 High-performance liquid chromatography (HPLC) analysis

HPLC was performed to identify and quantify the content of [6]-gingerol and [6]-shogaol in the GE. The compound of GE was analyzed using an Agilent 1260 Infinity II HPLC and UV detector. The conditions for the [6]-gingerol and [6]-shogaol (FUJIFILM Wako Pure Chemical Corporation, Osaka, Japan) analysis conditions are shown in Table 2. A stock solution of [6]-gingerol and [6]-shogaol in a standard was prepared in methanol. The quantification of the extract was calculated as the ratio of the peak area analyzed in the extract to the peak area of the standard product and was expressed as an average after two analyses.

**TABLE 2:** Analytical conditions of HPLC for [6]-gingerol and [6]-shogaol analysis.

Condition						
Treatment	[6]-gingerol			[6]-shogaol		
Column	YMC Triart C18 (250 × 4.6 mm, 5 μm)			YMC Triart C18 (250 × 4.6 mm, 5 μm)		
Flow rate	0.5 mL/min			0.5 mL/min		
Injection volume	10 µL			10 µL		
UV detection	280 nm			280 nm		
Run time	90 min			90 min		
Gradient	Time	%DW	%ACN	Time	%DW	%ACN
	0	55	45	0	55	45
	20	55	45	20	55	45
	25	60	40	25	60	40
	45	60	40	45	60	40
	50	100	0	50	100	0
	70	100	0	70	100	0
	75	55	45	75	55	45
	90	55	45	90	55	45

### 2.9 Statistical analysis

**FIGURE 2:**
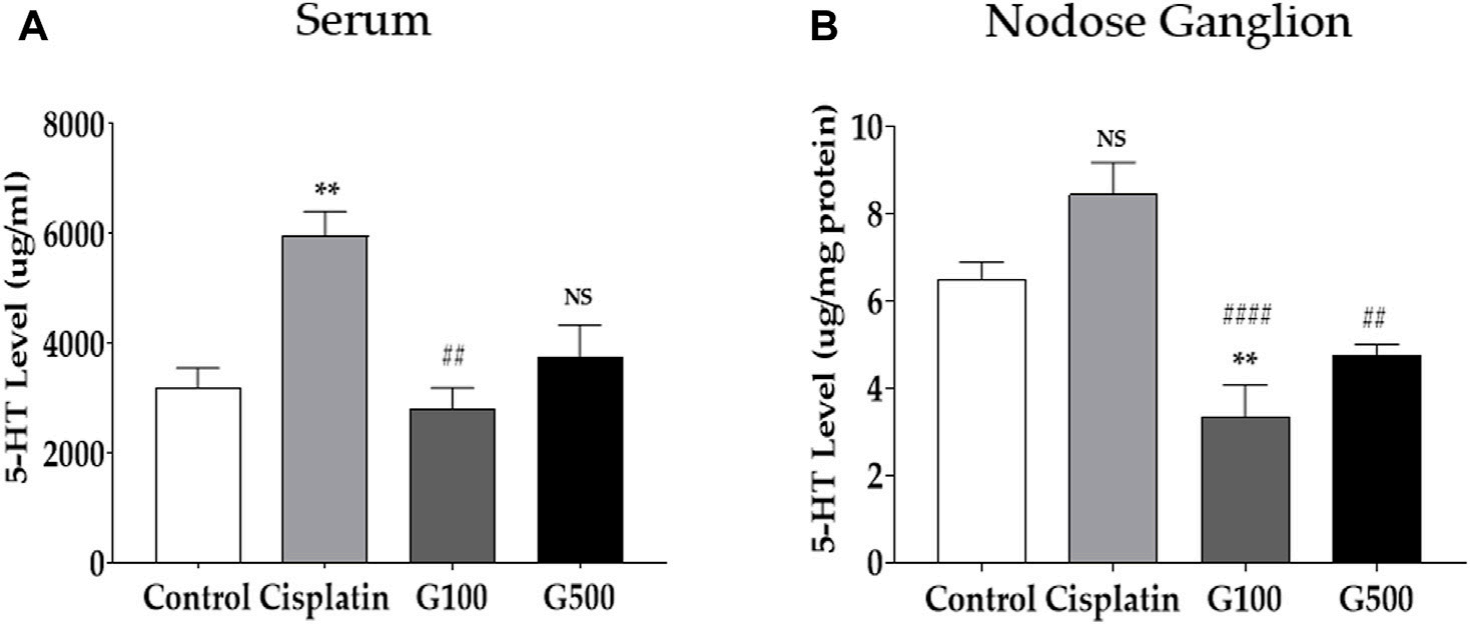
The quantification of 5-HT level in the serum and the nodose ganglion by using the ELISA method. 5-HT quantification in serum **(A)** and nodose ganglion **(B)** 48 h after administration of 6 mg/kg of cisplatin, 100 and 500 mg/kg of GE in rats. Control group rats received normal saline (i.p.) and DW (p.o.) as controls for cisplatin and GE, respectively. NS; non-significant, G100; ginger extract 100 mg/kg, G500; ginger extract 500 mg/kg. All experiments were performed in triplicate and data are presented as mean ± SEM. ** *p* < 0.01 vs. Control, ## *p* < 0.01, #### *p* < 0.0001 vs. Cisplatin, with one-way ANOVA followed by Tukey’s post-test for multiple comparisons.

**FIGURE 3:**
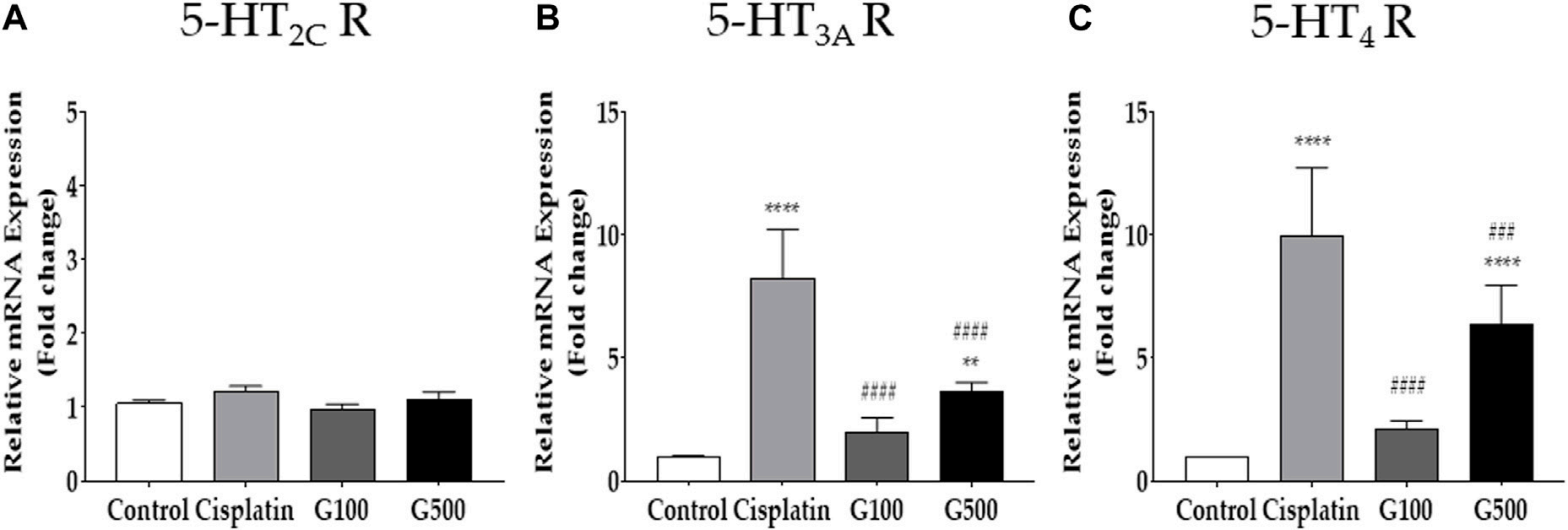
The relative gene expression of 5-HT receptors in the nodose ganglion measured by qRT-PCR. The relative gene expression of 5-HT_2C_ **(A)**, 5-HT_3A_ **(B)**, and 5-HT_4_ **(C)** receptors in the nodose ganglion 48 h after 6 mg/kg of cisplatin and 100 and 500 mg/kg of GE administration in rats. Control group rats received normal saline (i.p.) and distilled water (p.o.) as controls for cisplatin and GE, respectively. G100; ginger extract 100 mg/kg, G500; ginger extract 500 mg/kg. All experiments were performed in triplicate and data are presented as mean ± SEM. ** *p* < 0.01, **** *p* < 0.0001 vs. Control, ### *p* < 0.001, #### *p* < 0.0001 vs. Cisplatin, with two-way ANOVA followed by Tukey’s post-test for multiple comparisons.

**FIGURE 4:**
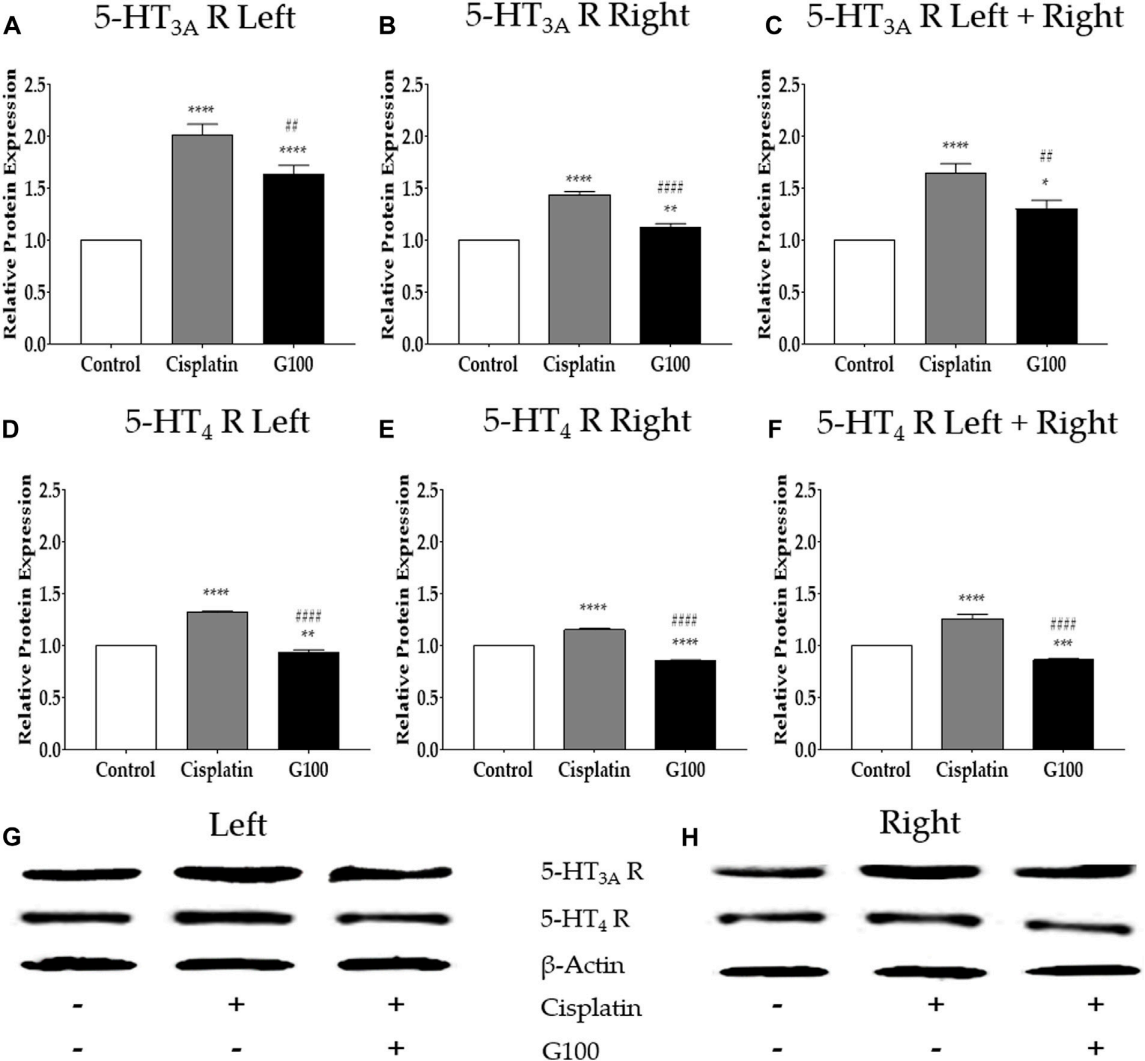
The relative protein expression of 5-HT_3A_ and _4_ receptors in nodose ganglion determined by western blot assay. The relative protein expression of 5-HT_3A_ and _4_ receptors in nodose ganglion at 48 h after administration of 6 mg/kg of cisplatin and 100 mg/kg of GE in rats. The results obtained from the left nodose ganglion **(A, D)**, right nodose ganglion **(B, E)**, sum of left and right nodose ganglia **(C, F)**, and western blot band images **(G, H)**. Control group rats received normal saline (i.p.) and distilled water (p.o.) as controls for cisplatin and GE, respectively. G100; ginger extract 100 mg/kg. All experiments were performed in triplicate and data are presented as mean ± SEM. * *p* < 0.05, ** *p* < 0.01, *** *p* < 0.001, **** *p* < 0.0001 vs. Control, ## *p* < 0.01, #### *p* < 0.0001 vs. Cisplatin, with one-way ANOVA followed by Tukey’s post-test for multiple comparisons.

**FIGURE 5:**
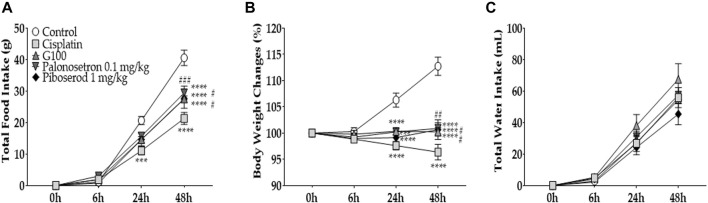
The effect of 5-HT_3A_ and _4_ receptors antagonists on cisplatin-induced anorexia in rats. The effect of palonosetron, piboserod, and 100 mg/kg of GE on cisplatin-induced anorexia in rats. Palonosetron and piboserod were used as antagonists for 5-HT_3A_ and _4_ receptors, respectively. Total intake of foods **(A)**, changes in body weight **(B)**, and water intake **(C)** were assessed. The dose of cisplatin 6 mg/kg was administered intraperitoneally to all four groups except the control group (i.e., cisplatin, G100, palonosetron, and piboserod). Control group rats received normal saline (i.p.) and distilled water (p.o.) as controls for cisplatin and GE, respectively. Cisplatin group was treated with distilled water (p.o.) as a control to GE. G100; ginger extract 100 mg/kg. All experiments were performed in triplicate and data are presented as mean ± SEM. *N* = 7 each control, cisplatin, G100, and piboserod group, *N* = 14 palonosetron group. *** *p* < 0.001, **** *p* < 0.0001 vs. Control, # *p* < 0.05, ## *p* < 0.01, ### *p* < 0.001 vs. Cisplatin, with two-way ANOVA followed by Tukey’s post-test for multiple comparisons.

Data are presented as the mean ± standard error of the mean (S.E.M.). Statistical analysis was performed by using Prism 7.0 Software (GraphPad Software Inc., San Diego, CA, United States). For comparisons, one-way ANOVA or two-way ANOVA with by Tukey’s post-tests were performed. One-way ANOVA were used for ELISA (Figure 2) and western blot (Figure 4) analysis. Two-way ANOVA was used for behavioral experiments (Figures 1, 5, 7) and qRT-PCR (Figure 3) analysis. *p < 0.05* was considered to indicate a statistically significant difference.

## 3 Results

### 3.1 The effect of ginger extract in cisplatin-induced acute anorexia in rats

To assess the effect of cisplatin on food intake, the amount of food consumed was measured at 6, 24, and 48 h following the injection after cisplatin injection (Figure 1A). Cisplatin was intraperitoneal injected once at the 0 h time point. The results show that 2 mg/kg and 6 mg/kg cisplatin significantly decreased food intake in rats 48 h after the injection (Figure 1B). This effect was more significant at 6 mg/kg than at 2 mg/kg. Furthermore, both body weight (Figure 1C) and water intake (Figure 1D) were significantly decreased compared to the control group at 24 and 48 h after a single cisplatin injection. However, body weight and water intake decreased only at 6 mg/kg, but not at 2 mg/kg cisplatin treated group. Therefore, further experiments were conducted using 6 mg/kg cisplatin. To assess the effect of ginger extract (GE) on cisplatin-induced acute food intake, two different doses of GE (100 and 500 mg/kg) were orally administered to rats injected with 6 mg/kg cisplatin (i.p.) (Figures 1A, E). The results show that both doses significantly alleviated the decreased food intake in rats at 48 h but not at the 24 h time point. However, body weight changes (Figure 1F) and total water intake (Figure 1G) demonstrated significant differences only in the 100 mg/kg GE group; 500 mg/kg-treated rats showed no significant differences compared to the cisplatin-treated group. GE treatment in naïve rats did not change the total food intake, body weight, or water intake compared to its control (Supplementary Figure S1).

### 3.2 GE decreases 5-HT in the serum and the nodose ganglion of the vagus nerve

As GE has been reported to affect the serotonergic system (Molahosseini et al., 2016; Lee et al., 2021; Kim et al., 2022; Min et al., 2022), enzyme-linked immunosorbent assay (ELISA) was performed to quantify the levels of 5-HT in the serum (Figure 2A) and nodose ganglia (Figure 2B) of cisplatin-induced anorexic rats. The serum was obtained via cardiac puncture. The left and right nodose ganglia and serum were obtained from rats 48 h after 6 mg/kg cisplatin (i.p.) and 100 and 500 mg/kg GE (p.o.) administration. Serum 5-HT levels increased after cisplatin treatment, whereas no change was observed in the nodose ganglion. However, oral treatment with 100 and 500 mg/kg GE reduced 5-HT levels in rats’ serum and nodose ganglion. Furthermore, to evaluate whether GE treatment (Supplementary Figure S2) or 2 mg/kg of cisplatin (Supplementary Figure S3) alone could change the serotonergic system after 48 h in rats, the serum 5-HT and 5-HT receptors in the nodose ganglia were also measured. However, both the serum 5-HT and serotonergic receptors remain unchanged.

### 3.3 GE prevents the upregulated gene expression of 5-HT receptors in the nodose ganglion of the vagus nerve in rats

The role of 5-HT receptors in the nodose ganglion in cisplatin-induced acute anorexia was assessed by qRT-PCR (Figure 3). Both the left and right nodose ganglia were obtained from rats 48 h after cisplatin (i.p.) and GE (p.o.) administration. The results showed that the levels of 5-HT_2C_ receptor did not change after cisplatin injection, whereas the expression of 5-HT_3A_ and _4_ receptors significantly increased. Oral treatment with 100 and 500 mg/kg GE alleviated the increased expression of 5-HT_3A_ and _4_ receptors in the nodose ganglion. The 5-HT_2C_ receptor levels remained unchanged after GE administration.

### 3.4 GE decreases the protein expression of 5-HT_3A_ and _4_ receptors in the nodose ganglion of the vagus nerve

To assess whether the changes in gene expression following cisplatin and GE treatment were related to changes in protein expression, western blot analysis was conducted on the nodose ganglion of rats. The results show that 48 h after cisplatin injection, the protein changes in 5-HT_3A_ and _4_ receptors in the nodose ganglion were significantly increased; however, 100 mg/kg GE significantly downregulated it (Figure 4). To observe whether there was a difference in receptor expression between the left and right nodose ganglia, data analyses were conducted separately (Figures 4A, B, D, E) and together (Figures 4C, F). However, in all six analyses, significant differences were observed, as cisplatin induced the upregulation of 5-HT_3A_ and _4_ receptors, whereas GE induced the downregulation of the receptor. Altogether, these results in protein expressions validate the results obtained from the qRT-PCR experiments. The effects of 5-HT_3A_ and _4_ receptors antagonists on cisplatin-induced acute anorexia.

### 3.5 The effects of 5-HT_3A_ and _4_ receptors antagonists on cisplatin-induced acute anorexia

To further confirm that 5-HT_3A_ and _4_ receptors play an important role in the anti-anorexic effect, palonosetron and piboserod 5-HT_3A_ and _4_ receptor antagonists, respectively, were injected into cisplatin treated rats (Figure 5). For comparison, 100 mg/kg of GE was administered. The dose of palonosetron (0.1 mg/kg) and piboserod (1 mg/kg) was set according to previous studies (Armstrong et al., 2006; Beattie et al., 2008; De Jonghe and Horn, 2009; Mine et al., 2013; Darmani et al., 2014; Darmani et al., 2015). The results show that palonosetron (i.p.) and piboserod (i.p.) could alleviate the decrease in food intake (Figure 5A) and weight loss (Figure 5B) similarly to that of 100 mg/kg of GE. The groups had no significant difference in the total water intake (Figure 5C).

### 3.6 Identification and quantification of [6]-gingerol and [6]-shogaol in GE

**FIGURE 6:**
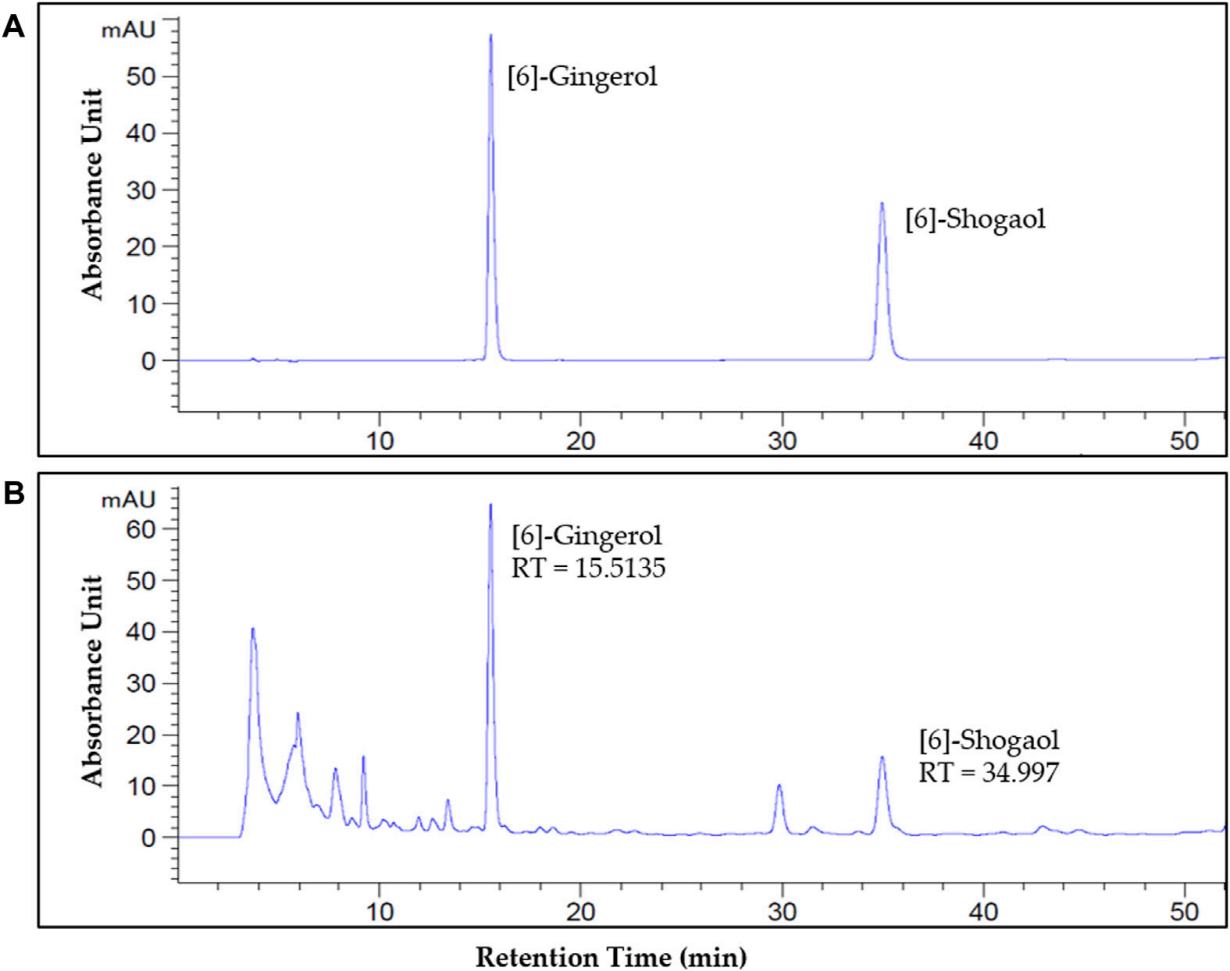
Identification and quantification of [6]-gingerol and [6]-shogaol in GE by high-performance liquid chromatography (HPLC). The peak of [6]-gingerol and [6]-shogaol in the standard **(A)** and GE **(B)**. The standard ultraviolet detection wavelength was set at 280 nm. The retention time (RT) of [6]-gingerol and [6]-shogaol was about 15.5 min and 34.9 min, respectively. The *X*-axis reports the retention time (RT), and the *Y*-axis the absorbance unit (AU).

To identify and quantify [6]-gingerol and [6]-shogaol, two major components of ginger, HPLC was conducted (Figure 6). The retention times (RT) of [6]-gingerol and [6]-shogaol were approximately 15.5 min and 34.9 min, respectively. The RT and spectrum absorbance unit (AU) of [6]-gingerol and [6]-shogaol in the standard (Figure 6A) and GE solutions were consistent (Figure 6B). The contents of [6]-gingerol and [6]-shogaol were 4.12% and 2.15%, respectively, in the 80% ethanol GE.

### 3.7 The effects of [6]-gingerol and [6]-shogaol on cisplatin-induced acute anorexia

**FIGURE 7:**
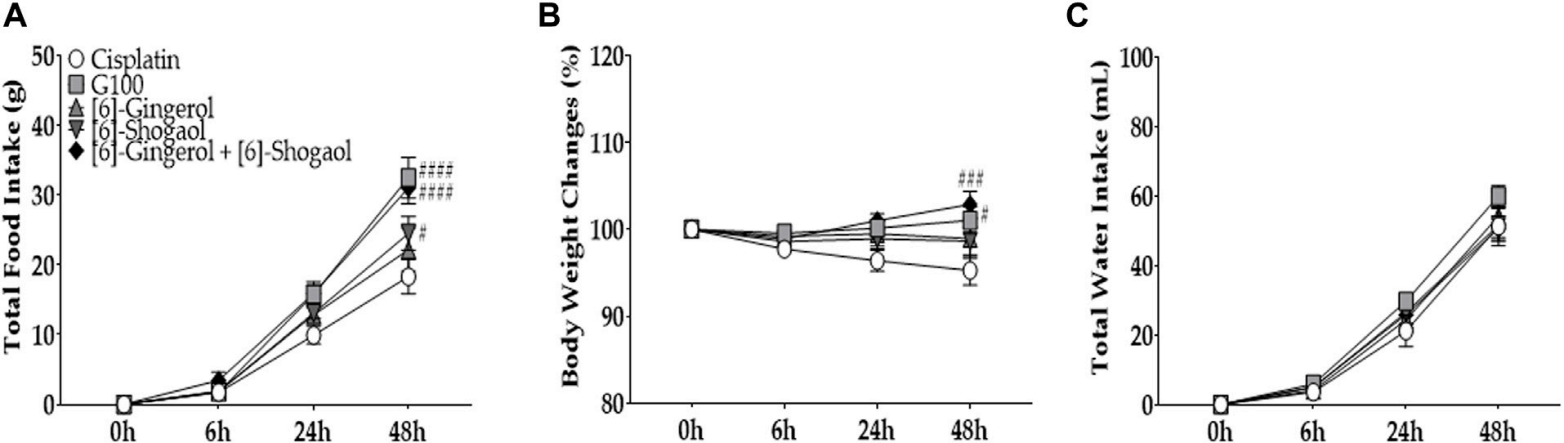
The effect of [6]-gingerol and [6]-shogaol on cisplatin-induced anorexia in rats. Total intake of foods **(A)**, changes in body weight **(B)**, and water intake **(C)** are presented. 6 mg/kg of cisplatin was injected intraperitoneally in five groups (i.e., cisplatin, G100, [6]-sho, [6]-gin, and [6]-sho + [6]-gin). In GE group rats, 100 mg/kg of GE was administered orally. In the [6]-gingerol and [6]-shogaol group, 4.12 mg/kg and 2.15 mg/kg of [6]-gingerol and [6]-shogaol were administered in cisplatin-injected rats, respectively. As a control, the cisplatin group (Cisplatin) was treated with distilled water (p.o.). G100; ginger extract 100 mg/kg, [6]-gin; [6]-gingerol, [6]-sho; [6]-shogaol, [6]-sho + [6]-gin; [6]-shogaol and [6]-gingerol. All experiments were performed in triplicate and data are presented as mean ± SEM. N = 8 for cisplatin, G100, and [6]-sho + [6]-gin groups, *N* = 12 for [6]-sho and [6]-gin groups. # *p* < 0.05, ### *p* < 0.001, #### *p* < 0.0001 vs. Cisplatin, with two-way ANOVA followed by Tukey’s post-test for multiple comparisons.

To assess the role of the two major components of GE, [6]-gingerol and [6]-shogaol were injected intraperitoneally into cisplatin-treated rats (Figure 7A). As according to HPLC analysis, 4.12% and 2.15% of [6]-gingerol and [6]-shogaol, respectively, were shown to compose 100 mg/kg of GE, 4.12 mg/kg of [6]-gingerol and 2.15 mg/kg of [6]-shogaol were injected in rats. The results showed that when [6]-gingerol or [6]-shogaol was administered separately, no difference was observed in rats, whereas co-administration of the two components resulted in an anti-anorexic effect similar to that of 100 mg/kg GE. Although body weight also changed (Figure 7B), there was no significant difference in water intake (Figure 7C).

## 4 Discussion

In this study, we showed that 6 mg/kg cisplatin reduced food intake, body weight, and water intake 48 h after its injection in rats. This acute anorexia was reversed by oral administration of 100 mg/kg and 500 mg/kg of ginger extract (GE). The 5-HT receptors in the nodose ganglia were shown to play a critical role, as they were significantly upregulated and downregulated after cisplatin and GE treatments, respectively. To the best of our knowledge, this is the first study to assess the effects of GE on cisplatin-induced anorexia.

In our study, 6 mg/kg cisplatin caused more severe signs of decreased appetite than 2 mg/kg cisplatin. Therefore, 6 mg/kg was administered throughout the experiments. According to the dose injected in humans, 6 mg/kg cisplatin is the closest concentration in rats; in humans, the cisplatin treatment range is 35 mg/m^2^ based on 60 kg of humans, which is 6 mg/kg in 200 g of rats based on body surface area (BSA) (Reagan‐Shaw et al., 2008).

Ginger (*Zingiber officinale* Roscoe) is a plant from the Zingiberaceae family mainly cultivated in Asia (Yadav et al., 2004). Several studies have reported analgesic, anti-inflammatory, antioxidant, and antimicrobial effects of ginger (Kikuzaki and Nakatani, 1993; Ali et al., 2008). As an anti-inflammatory agent, 500 mg/kg GE has been reported to significantly lower serum prostaglandin-E2, which is a physiologically active substance associated with inflammation (Thomson et al., 2002). Furthermore, breast tumorigenesis in mice was significantly suppressed when mice had free access to GE (Nagasawa et al., 2002). In this study, 100 mg/kg GE significantly increased cisplatin-treated rats’ food intake and body weight. Higher doses of GE (i.e., 500 mg/kg) also succeeded in increasing food intake; however, they failed to augment body weight or water intake. Although the reasons for these differences are difficult to understand, GE has been reported to enhance thermogenesis and has been suggested to play a potential role in weight management (Mansour et al., 2012; Macit et al., 2019). Thus, increasing the GE dose may enhance thermogenesis and prevent weight gain in treated rats. Moreover, although 500 mg/kg GE was effective in decreasing the 5-HT level and the gene expression of 5-HT_3A_ and _4_ receptors, 100 mg/kg was more efficient (vs. cisplatin group: 0.0024 (100 mg/kg) vs. 0.2073 (500 mg/kg); 0.0001 (100 mg/kg) vs. 0.0074 (500 mg/kg); <0.0001 (100 mg/kg) vs. < 0.0001 (500 mg/kg); <0.0001 (100 mg/kg) vs. 0.0001 (500 mg/kg)) suggesting this differences may have somehow affected the metabolism and prevent the increase in the body weight. However, further studies are required to elucidate the role of GE in weight management.

In addition, GE successfully decreased 5-HT levels in cisplatin-treated rats’ blood and nodose ganglia. The 5-HT receptors in the nodose ganglia were also altered; they were upregulated following the injection of cisplatin and significantly downregulated after treatment with GE. Sensory neurons of the nodose ganglion are pseudo-unipolar, innervate various organs in the thorax and abdomen, and send signals to different regions of the nucleus of the solitary tract (NTS) of the medulla (Tonini, 1995). Mechanical distension and chemical/hormonal signals such as leptin, ghrelin, cholecystokinin (CCK) are known to affect the vagus nerve (Cork, 2018). In addition, cardiovascular, respiratory, and gastrointestinal information is transmitted to the brain (Zhuo et al., 1997). Among the neurons sending projections to the NTS, 5-HT immunoreactive neurons have been identified in the nodose ganglia (Nosjean et al., 1990). In a study conducted by Wu et al. (2005) luminal perfusion of 5-HT significantly increased the number of cells expressing c-Fos in nodose ganglia, whereas granisetron, a 5-HT_3_ receptor antagonist, reduced the increase in the expression of nodose neuronal c-Fos. Although the role of 5-HT receptors in the brain of anorexic rodents has been demonstrated in some studies (Haleem, 2012), their role in the nodose ganglia has not been clearly demonstrated. Both the left and right ganglia receive signals from the branches of the stomach. The left innervates the dorsal surface of the stomach, whereas the right nodose ganglion receives signals from the ventral surface of the stomach (Berthoud and Neuhuber, 2000; Waise et al., 2018). In our study, the protein expression of 5-HT_3A_ and _4_ receptors were analyzed separately in the left and right nodose ganglia (Figure 4) to see whether differences exist between the two ganglia. Both protein expression increased and decreased significantly after cisplatin and GE administration, but no significant difference between left and right was observed.

In this study, 6 mg/kg cisplatin significantly increased the gene and protein expression of 5-HT_3A_ and _4_, but not _2C_ receptors, and GE, which significantly increased food intake and downregulated the expression of both 5-HT_3A_ and _4_ receptors in cisplatin-treated rats. Although some studies have reported the role of 5-HT_2C_ receptor in feeding behaviors (Sargent et al., 1997; Halford and Harrold, 2012), their role in the peripheral nervous system needs further investigation, as the distribution of 5-HT_2C_ receptor has been shown to be more concentrated in the central nervous system (Hoyer et al., 2002). In addition, although dietary intake increased in 5-HT_2C_ receptor-knockout mice, this action was not reproduced when the 5-HT_2C_ receptor antagonist SB242084 was injected, suggesting that further research is needed to clarify its role in cisplatin-induced anorexia (Bickerdike et al., 1999). The GE did not alter both the feeding behaviors and serotonergic system when treated in naïve rodents (Supplementary Figures S1, S2). These results show that when GE is given orally in naïve rats, its 5-HT modulatory effect is not induced. Furthermore, although in our study, the negative effect of GE has not been assessed, GE is reported be relatively safe compared to other drugs as daily administrations of 500, 1000, or 2000 mg/kg of GE for multiple days induced no unusual clinical signs (Rong et al., 2009).

In this study, 2 mg/kg of cisplatin did not alter the 5-HT level in the serum compared to control. Furthermore, 5-HT_3A_ and _4_ receptors did not also change compared to control on the contrary to 6 mg/kg of cisplatin where both 5-HT and its receptors expression altered. In *in vivo* tests, 2 mg/kg cisplatin decreased the 48 h total food intake, whereas body weight and water intake remain unchanged. Although it is difficult to understand the precise reasons of the difference, we think that other substances than 5-HT such as the ghrelin may have also acted in the effect of cisplatin (Hattori et al., 2013). However, to decrease the appetite in larger volume and induce body weight changes as in 6 mg/kg treated group rats, the role of 5-HT appears to be critical.

In our next experiment, 5-HT_3A_ and _4_ receptors antagonists were injected to confirm the role of 5-HT_3A_ and _4_ receptors in cisplatin-induced anorexia. Palonosetron and piboserod are 5-HT_3A_ and _4_ receptors antagonist, respectively. The results show that they significantly alleviated the decrease in food intake and body weight similarly to 100 mg/kg GE treated rats. Palonosetron is a 5-HT_3_ receptor antagonist and has been reported to have a high binding affinity, greater potency, and longer potency with 5-HT_3_ receptor (Yang and Scott, 2009). It has a 100-fold greater binding affinity to the 5HT_3_ receptor (Eisenberg et al., 2003) and has a long half-life of 40 h compared to other 5-HT_3_ receptor antagonist (i.e. 3-9 h) (Eisenberg et al., 2004; Aapro, 2007). Piboserod (SB207266) is an antagonist of 5-HT_4_ receptor and has been known to be a very powerful and long-acting 5-HT_4_ receptor antagonist with about 1,000-fold greater binding affinity for 5-HT_4_ than other 5-HT receptors (Wardle et al., 1996). Therefore, referring to the various *in vivo* studies mentioned above, the effective concentration and administration methods of palonosetron and piboserod were reviewed (Armstrong et al., 2006; Beattie et al., 2008; De Jonghe and Horn, 2009; Mine et al., 2013; Darmani et al., 2014; Darmani et al., 2015).

Finally, among the various components of GE, [6]-gingerol and [6]-shogaol are reported to be the two major components, constituting 4.12% and 2.15% of GE, respectively. However, when the two components were administered separately, no significant effect was observed, whereas the co-administered group demonstrated a significant effect against cisplatin-induced anorexia. Both [6]-gingerol and [6]-shogaol have antagonistic effects on serotonergic system (Abdel-Aziz et al., 2006; Pertz et al., 2011; Jin et al., 2014). These results suggest that the action of only either [6]-gingerol or [6]-shogaol alone may not be sufficient to affect the effect of cisplatin, and both components should be administered to prevent the development of acute anorexia.

In conclusion, our results show that oral administration of 100 and 500 mg/kg GE could significantly alleviate acute anorexia induced in 6 mg/kg cisplatin-treated rats and that 5-HT and its receptors (i.e., 5-HT_3A_ and _4_ receptors in the nodose ganglia) play an important role in the action of GE. Further research is also required to clearly understand the underlying mechanism of action of GE, but this effect can be attributed to the action of two major sub-components of GE: [6]-gingerol and [6]-shogaol. Our results suggest that GE should be considered as an option for attenuating cisplatin-induced anorexia. However, more well-designed experimental studies should be conducted to completely understand the role of GE in chemotherapy-induced anorexia. Future studies should focus on the brain appetite regulatory areas such as NTS and paraventricular nucleus (PVN) and appetite regulatory substances such as the ghrelin and leptin.

## Data Availability

The original contributions presented in the study are included in the article/Supplementary Material, further inquiries can be directed to the corresponding author.

## Supplementary material

SUPPLEMENTARY FIGURE S1 The effect of GE 100 mg/kg on total food intake, body weight changes, and water intake in naive rats. The amount of total food consumed **(A)**, body weight changes **(B)**, and total water intake **(C)** were measured at 6, 24, and 48 h time points after single GE injection. Control group rats received only distilled water (p.o) as a control to GE. G100; ginger extract 100 mg/kg. Data are presented as the mean ± standard error of the mean (S.E.M.). *N* = 6 each group. *P* < 0.05 was considered to indicate a statistically significant difference. For comparisons, two-way ANOVA with by Tukey’s post-tests were performed.

SUPPLEMENTARY FIGURE S2 Assessments of the level of 5-HT, 5-HT_3A_ and 5-HT_4_ receptors changes after GE administration in naive rats. 100 mg/kg of GE was orally treated in rats. Rats were scarified 48 h after the injection for 5-HT level **(A)** and 5-HT_3A_ **(B)** and 5-HT_4_ **(C)** receptors gene expression change quantification in the serum and nodose ganglia, respectively. No significant difference was shown between the groups. Control: DW injected rats; Control+G100; DW and ginger extract 100 mg/kg treated group. Data are presented as the mean ± SEM. *N* = 4 each group. *P* < 0.05 was considered to indicate a statistically significant difference. For comparisons, one-way ANOVA or two-way ANOVA with by Tukey’s post-tests were performed. One-way ANOVA were used for ELISA (A). Two-way ANOVA was used for qRT-PCR **(B, C)** analysis.

SUPPLEMENTARY FIGURE S3 Evaluation of 5-HT and 5-HT_3A_ and 5-HT_4_ receptors changes after cisplatin and GE administrations. Cisplatin (2 mg/kg) and GE (100 mg/kg) were administered intraperitoneally and orally in rats, respectively. 5-HT quantification in serum **(A)** and 5-HT_3A_ **(B)** and 5-HT_4_ **(C)** receptors gene expression in nodose ganglion 48 h after administration of cisplatin and GE. Control group rats were injected with normal saline and DW as control to cisplatin and GE, respectively. G100; ginger extract 100 mg/kg. Data are presented as the mean ± SEM. *N* = 4 each group. P < 0.05 was considered to indicate a statistically significant difference. For comparisons, one-way ANOVA or two-way ANOVA with by Tukey’s post-tests were performed. One-way ANOVA were used for ELISA **(A)**. Two-way ANOVA was used for qRT-PCR **(B, C)** analysis.
