# Supplementary figures and images for: The effect of ginger extract on cisplatin-induced acute anorexia in rats
Source: Front Pharmacol. 2023 Nov 9;14:1267254. doi: 10.3389/fphar.2023.1267254 (PMC10665510; doi:10.3389/fphar.2023.1267254)

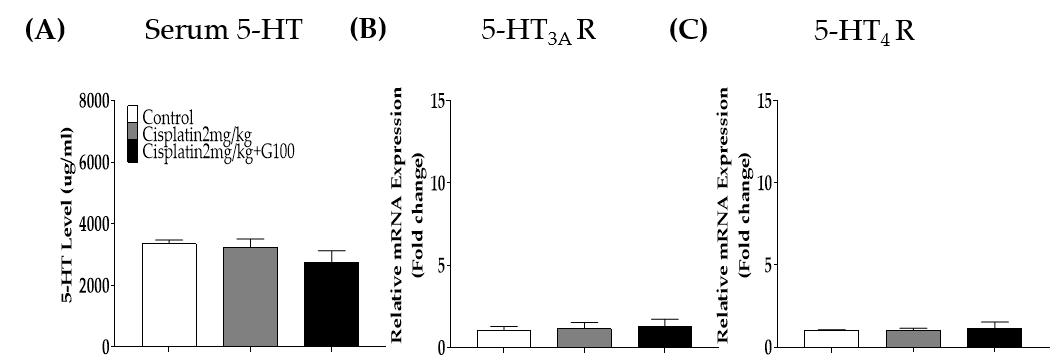

Supplement: Supplementary file 1 [file Image3.TIF]

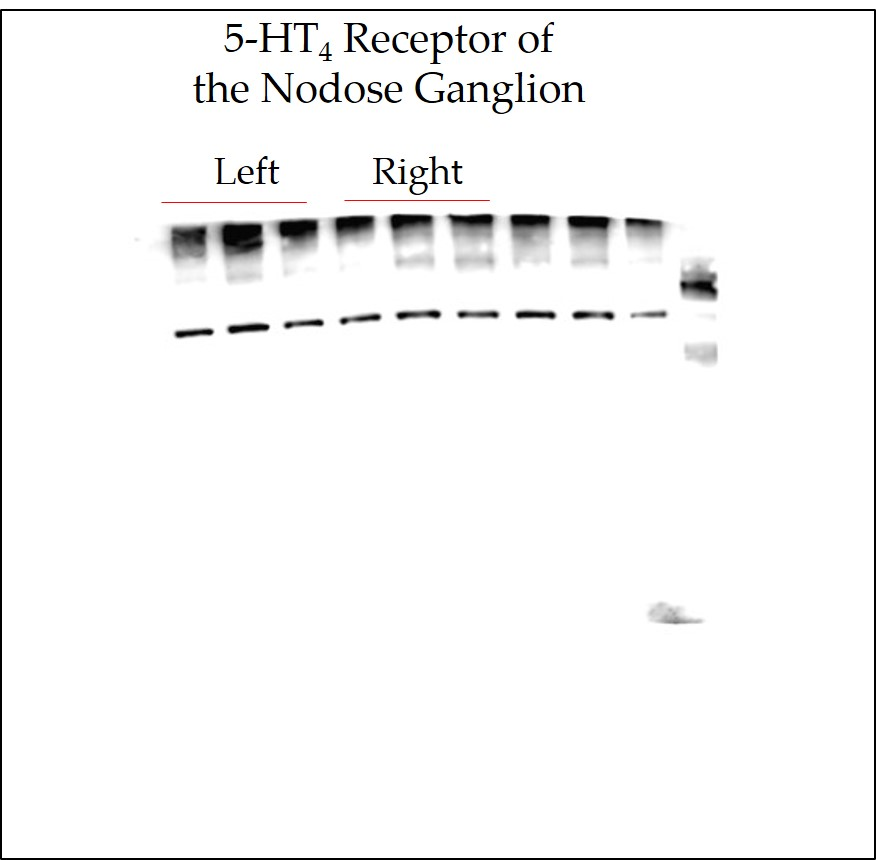

Supplement: Supplementary file 2 [file Image5.TIFF]

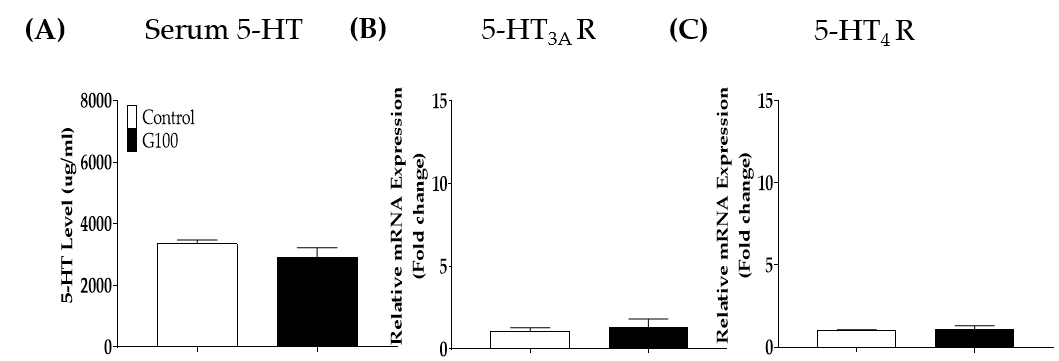

Supplement: Supplementary file 3 [file Image2.TIF]

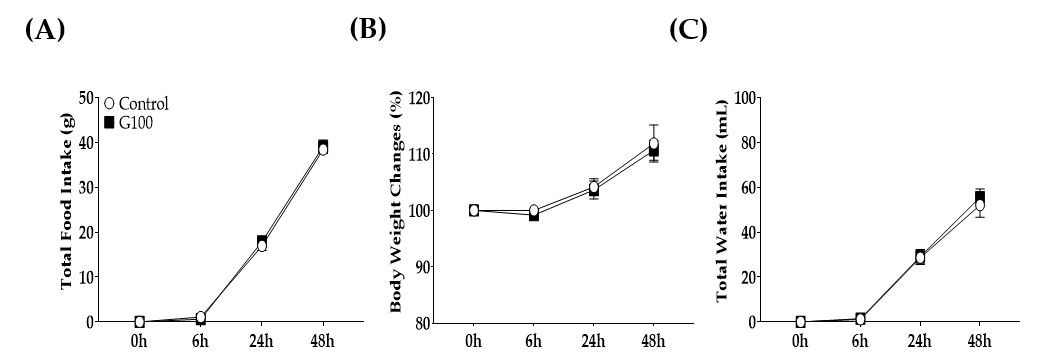

Supplement: Supplementary file 4 [file Image1.TIF]

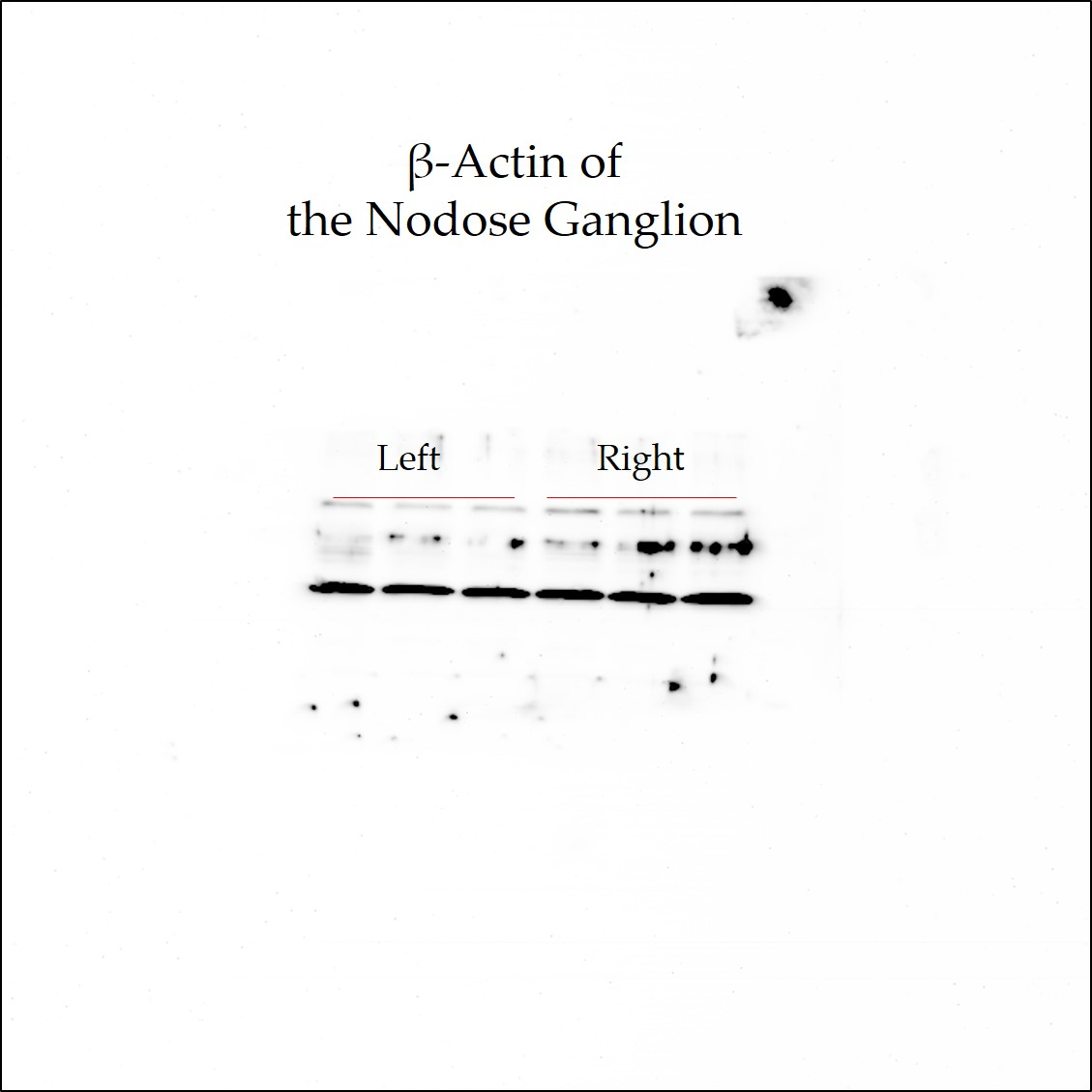

Supplement: Supplementary file 5 [file Image6.TIFF]

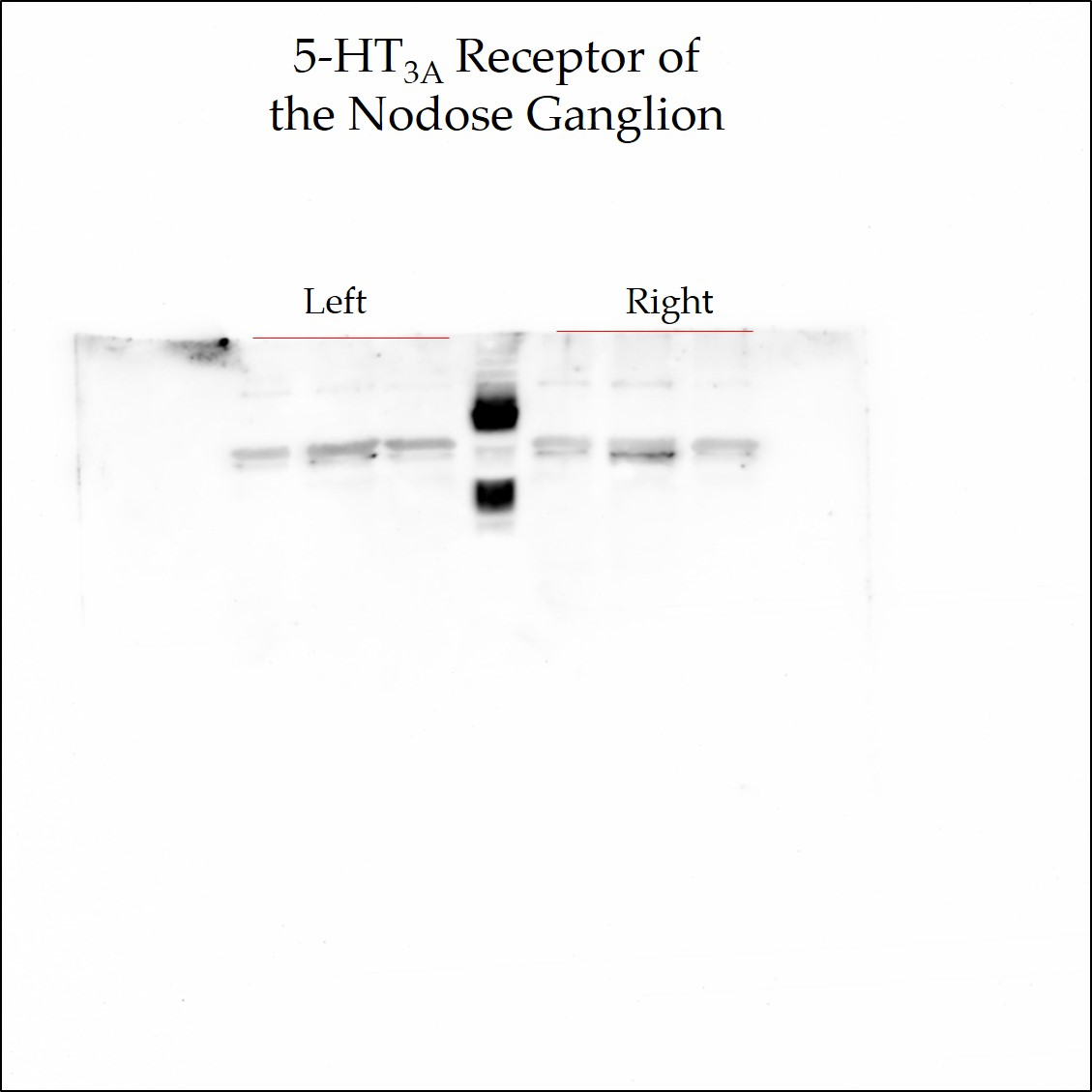

Supplement: Supplementary file 6 [file Image4.TIFF]
